# Plasma pTau217 Predicts AD and Disease Progression in a Diverse Cohort

**DOI:** 10.1002/alz.093105

**Published:** 2025-01-09

**Authors:** James J. Lah, Blaine Russell Roberts, Caroline M Watson, Erik C.B. Johnson, Chadwick M Hales, Deqiang Qiu, Thomas S. Wingo, Nicholas T Seyfried, Andreas Jeromin, Allan I. Levey

**Affiliations:** ^1^ Emory University School of Medicine, Atlanta, GA USA; ^2^ ALZpath, Inc., Carlsbad, CA USA

## Abstract

**Background:**

Plasma pTau217 detects AD pathology and show longitudinal changes dependent on baseline pathology. However, previous studies have been restricted to cohorts with limited diversity. We evaluated performance of plasma pTau217 in detecting AD and predicting disease progression in a diverse cohort from the Emory Goizueta ADRC.

**Method:**

We analyzed plasma pTau217 levels using AlzPath antibody and the Simoa HD platform. The cohort includes 410 Controls, 100 MCI, and 249 AD cases with research consensus diagnoses, 38.4% self‐identified African American (AA) individuals, with a subset of 478 cases with CSF AD biomarkers. Baseline plasma samples were analyzed for accuracy in classifying diagnosis and predicting progression (i.e., Control to MCI or MCI to AD). Paired samples for 244 participants were used to determine longitudinal changes.

**Result:**

In baseline samples, median pTau217 levels were 0.318 pg/ml in Controls, 0.678 pg/ml in MCI, and 1.151 pg/ml in AD. Differences between Control, MCI, and AD were significant (p<0.0001 for all pairwise comparisons) for the entire cohort and within self‐identified AA and White subgroups. Group comparison by race revealed lower pTau217 in AA vs. White individuals among Controls (0.297 vs. 0.351; p<0.0001) and AD (1.046 vs. 1.266; p<0.05) groups. In longitudinal samples, progressors had higher pTau217 at baseline compared to non‐progressors (Controls: 0.939 vs. 0.336, p<0.0001; MCI: 1.056 vs. 0.678, p=0.040). Plasma pTau217 increased across all longitudinal samples at 0.020 pg/ml/yr. Rate of increase in pTau217 was higher among those with AD at baseline compared to Control non‐progressors (0.077 vs. 0.017; p=0.035). No other group differences reached statistical significance but analysis was limited by small sample sizes.

**Conclusion:**

Baseline plasma pTau217 levels are different among Control, MCI, and AD cases for AA and White individuals, and higher baseline levels are associated with higher rate of longitudinal increase and predict disease progression. However, AA individuals collectively have lower pTau217 levels than White individuals regardless of disease status. Self‐identifies racial differences in plasma pTau levels must be addressed when using cut‐points for both research and clinical applications.

